# Methane Catalytic Combustion under Lean Conditions over Pristine and Ir-Loaded La_1−x_Sr_x_MnO_3_ Perovskites: Efficiency, Hysteresis, and Time-on-Stream and Thermal Aging Stabilities

**DOI:** 10.3390/nano13152271

**Published:** 2023-08-07

**Authors:** Catherine Drosou, Ersi Nikolaraki, Theodora Georgakopoulou, Sotiris Fanourgiakis, Vassilios T. Zaspalis, Ioannis V. Yentekakis

**Affiliations:** 1 Laboratory of Physical Chemistry and Chemical Processes, School of Chemical and Environmental Engineering, Technical University of Crete, 731 00 Chania, Crete, Greece; enikolaraki@tuc.gr (E.N.); theodorageorgakopoulou@gmail.com (T.G.); sfanourgiakis@tuc.gr (S.F.); 2 Department of Chemical Engineering, Aristotle University of Thessaloniki, 541 24 Thessaloniki, Greece; zaspalis@auth.gr; 3 Chemical Process and Energy Resources Institute, Center for Research and Technology Hellas (CPERI/CERTH), 570 01 Thermi, Thessaloniki, Greece; 4 Institute of GeoEnergy, Foundation for Research and Technology-Hellas (FORTH/IG), 731 00 Chania, Greece

**Keywords:** catalytic methane oxidation, LSM perovskites, iridium, thermal aging stability, lean conditions, hysteresis phenomena

## Abstract

The increasing use of natural gas as an efficient, reliable, affordable, and cleaner energy source, compared with other fossil fuels, has brought the catalytic CH_4_ complete oxidation reaction into the spotlight as a simple and economic way to control the amount of unconverted methane escaping into the atmosphere. CH_4_ emissions are a major contributor to the ‘greenhouse effect’, and therefore, they need to be effectively reduced. Catalytic CH_4_ oxidation is a promising method that can be used for this purpose. Detailed studies of the activity, oxidative thermal aging, and the time-on-stream (TOS) stability of pristine La_1−x_Sr_x_MnO_3_ perovskites (LS_X_M; X = % substitution of La with Sr = 0, 30, 50 and 70%) and iridium-loaded Ir/La_1−x_Sr_x_MnO_3_ (Ir/LS_X_M) perovskite catalysts were conducted in a temperature range of 400–970 °C to achieve complete methane oxidation under excess oxygen (lean) conditions. The effect of X on the properties of the perovskites, and thus, their catalytic performance during heating/cooling cycles, was studied using samples that were subjected to various pretreatment conditions in order to gain an in-depth understanding of the structure–activity/stability correlations. Large (up to ca. 300 °C in terms of T_50_) inverted volcano-type differences in catalytic activity were found as a function of X, with the most active catalysts being those where X = 0%, and the least active were those where X = 50%. Inverse hysteresis phenomena (steady-state rate multiplicities) were revealed in heating/cooling cycles under reaction conditions, the occurrence of which was found to depend strongly on the employed catalyst pre-treatment (pre-reduction or pre-oxidation), while their shape and the loop amplitude were found to depend on X and the presence of Ir. All findings were consistently interpreted, which involved a two-term mechanistic model that utilized the synergy of Eley–Rideal and Mars–van Krevelen kinetics.

## 1. Introduction

The replacement of traditional fossil fuels with cleaner and/or sustainable energy sources is currently imperative due to the recent global energy and environmental crisis. Regarding the global transition toward so-called “low carbon footprint energy technologies” and successful sustainability, humanity has exhibited an ever-increasing dependence upon natural gas (NG); gas is widely considered to be the ‘bridge fuel’ during this transition period [1,2,3,4]. NG typically contains ~85–95% methane [4,5,6]. The high efficiency of methane as a fuel, as well as the associated low emissions of NO_x_, CO, and particulate pollutants when producing thermal energy in urban tissues and industries, or when producing mechanical energy for vehicles, make it a greener alternative compared with other fossil fuels; this is because it produces reliable and affordable energy, and it promotes the development of other “green” energy sources [4]. In addition, its use in added-value chemical production [7,8,9], synthesis gas and/or H_2_ production (with the simultaneous recycling of CO_2_ emissions via dry reforming processes [10,11,12,13,14,15]), as well as its direct (internal) electrochemical conversion which produces electricity using solid oxide fuel cells (SOFCs) [16,17,18,19,20], are some additional important applications of CH_4_ in cleaner energy production technologies. However, as the use of methane in this plethora of applications expands, so do the emissions of unconverted methane, most of which have a low methane content of 0.1–1.0% [21,22,23,24,25,26,27,28]. Such emissions must be urgently and effectively mitigated given that CH_4_ is one of the main greenhouse gases; it has a greenhouse efficiency that is 25 times greater than CO_2_, otherwise, the climate benefits of its use could be offset [1,2,3].

As CH_4_ contains a very strong C–H bond (450 kJ/mol), its thermal combustion with air requires very high temperatures (ca. 1500 °C) compared with other HCs. Moreover, it simultaneously produces higher amounts of NO_x_ as by-products of the oxidation of N_2_ (found in the air) in the burners operating at such high temperatures [4,5]. Therefore, the catalytic oxidation of methane is considered advantageous for controlling its end-of-pipe emissions due to the relatively low operating temperatures required; this is associated with minimal NO_x_ emissions. However, it is still true that the strong C–H bond in the methane molecule makes its catalytic activation, and thus its oxidation, relatively more difficult compared with other hydrocarbons. In recent years, various families of materials have been proposed as catalysts for the deep (complete) oxidation of methane, emphasizing on the activity and thermal stability of the materials, as well as their dependence on the chemical composition of the materials and the preparation method used [4,5,21,22,23,24,25,26,27,28].

In this regard, catalysts based on noble metal (NM) nanoparticles that are dispersed on oxide or mixed oxide supports are typically more active than other families of materials, but they have the disadvantages of being priced at a high cost, and they tend to gradual deactivate during operation due to the aggregation of the noble metal nanoparticles [22,27,28]. Among NMs, iridium has shown quite an encouraging performance in emission control catalysis, which involves the oxidation of CO and volatile organic compounds (VOCs), de-NO_x_, and de-N_2_O reactions [29,30,31,32,33,34], as well as in CH_4_ reforming reactions [14,15,35,36], but their application in real systems is limited due to the high aggregation propensity of Ir nanoparticles under oxidizing conditions at elevated temperatures [37,38,39]. However, we have recently demonstrated that sensitive to agglomeration metal nanoparticles can be effectively stabilized through metal-support interactions induced by supports possessing a high oxygen storage capacity (OSC) and high oxygen ions mobility (labile lattice oxygen) [39,40,41]. A model that could consistently interpret these findings was developed. It was based on the effect of thermally driven O^2−^ ions moving from the supports onto catalyst NPs in order to create an O^δ−^ layer on their surfaces. Then, as a protective coat, it resists the main particle agglomeration mechanisms, namely, the Particle Migration and Coalescence (PMC) and Ostwald Ripening (OR) mechanisms [40,41].

On the other hand, mixed metal oxides, especially those of the perovskite class (ABO_3_), combine high thermal and chemical stabilities with the adequate activity of oxidation reactions, while keeping their cost relatively low [42,43,44,45,46]. Perovskites based on the combination of La and Mn in the A and B sites, respectively, are among the most popular materials in the family. The partial substitution of La^3+^ by Sr^2+^ in the perovskite structure (i.e., La_1−x_Sr_x_MnO_3_) can enhance the redox properties of the material by increasing the oxygen vacancies and the oxidation state of the B cation (Mn), introducing significant changes to their catalytic performance and their thermal stability [42,43,44,45,46,47,48,49]. Especially after the inspiring discovery of Nishihata et al. [50] and Tanaka et al. [51], who demonstrated the self-regenerative function of noble metal substituted perovskites, a phenomenon that was later called “redox exclusion” [52,53], as well as methods that determine the nanostructure of the perovskite itself, thus improving the surface area and other catalysis-related properties [54,55], new horizons were opened for the use of perovskites in various environmental- [54,56,57,58,59] and energy- [55,60,61,62,63,64] related catalytic processes, which resulted in great success.

However, the classical partial substitution of the A and/or B sites of an ABO_3_ perovskite with other cations, with the same or different valences, to obtain substituted (as called) A_1−y_A’yB_1−x_B’_x_O_3±δ_ perovskites, remains a popular methodology for controlling the performance of perovskites due to its high flexibility. The bulk, surface, and redox properties of the original ABO_3_ perovskite can be easily tailored on demand [43,44,45,46,47]. Emphasis is always placed on the oxygen storage capacity (OSC), oxygen ion mobility, and population of the surface oxygen vacancies (O defects) of the substituted perovskites; these properties play a key role in catalysis via oxides [56,57,58,59,60,61,62,63,64,65,66,67], as well as in catalysis via dispersed metal nanoparticles on oxide supports [13,14,15,68,69,70,71,72,73,74,75]. Regarding the latter, these properties are critical for the development of desirable metal–support interactions. It is therefore obvious why perovskites are a class of materials that attract high and ever-increasing interest with regard to heterogeneous catalysis; they are either used as pristine materials, exploiting their own advantageous characteristics for catalysis, or as effective supports for metal nanoparticles, as they endow them with favorable metal-support interactions.

In a previous study, the effect of the degree of the substitution of La by Sr on La_1−x_Sr_x_MnO_3_ perovskite and their counterpart, iridium-loaded catalysts (Ir/La_1−x_Sr_x_MnO_3_), was studied in CO oxidation with excess O_2_ reaction [30]. Both catalyst series were found to be effective in the reaction, with the Ir/La_1−x_Sr_x_MnO_3_ catalysts significantly outperforming their pristine La_1−x_Sr_x_MnO_3_ counterparts. Interesting inverse hysteresis phenomena were observed during heating/cooling cycles, depending on both the degree of substitutional Sr (x), and the pretreatment (pre-oxidation/pre-reduction) of the catalysts. The results were part of a project to decipher the effectiveness of perovskite-based catalysts, in combination with the relatively cheap noble metal, Ir, in reactions related to the control of NG-powered vehicle emissions. Traffic caused by NG-fueled vehicles is increasing rapidly (ca. 23 million worldwide, ranging from heavy-duty to light-duty cars, with an annual increase of 20%) [4]. Catalysis of CH_4_ combustion is also of particular interest because unburnt CH_4_ also exists in the exhaust stream of NG-powered engines and processes [21,22,23,24,25,26,27,28,64,65,66,67,68]. Complete methane combustion is also a useful side process in the very demanding application of natural-gas (NG) fueled gas-turbines (GT), as they operate alongside the catalytically stabilized hybrid combustion concept [76].

In the present work, lean CH_4_ combustion on pristine La_1−x_Sr_x_MnO_3_ (LS_X_M; X = 0, 30, 50 and 70% substitution of La with Sr) perovskites and their 2 wt% iridium loaded counterparts (Ir/LS_X_M) is comparatively studied in a temperature range of 400–970 °C. The impact of the degree of substitution of the A-site (La) of the perovskite with Sr, on its catalytic behavior is explored. This occurs after an exploration into the catalysts’ various pre-treatment protocols, such as pre-reduction, pre-oxidation, and thermal aging, which were implemented to obtain a complete overview of their CH_4_ combustion performance and how it correlates with the morphological characteristics and properties of the materials. Complex hysteresis phenomena recorded for the first time using this catalyst/reaction system during heating/cooling cycles were therefore consistently interpreted. Although LSM perovskites are possibly among the most studied materials for their catalytic performance in various reactions, not excluding some studies concerning the oxidation of CH_4_, the highly systematic activity and stability studies performed herein revealed new phenomena and findings that could be both of specific and general interest for catalysis research.

## 2. Materials and Methods

### 2.1. LSxM and Ir/LSxM Catalysts Synthesis

The unloaded and 2 wt% Ir loaded perovskite-type La_1−x_Sr_x_MnO_3_ (denoted hereafter as LSxM, where x = 0, 30, 50 and 70 expresses the % replacement of La with Sr in the perovskite formula) catalysts were prepared using the co-precipitation method described by Haron et al. [77]. The nitrate salts, La(NO_3_)_3_∙6H_2_O (VWR Chemicals, 99.9%), Sr(NO_3_)_2_ (Sigma Aldrich, St. Louis, MO, USA, 99.0%) and Mn(NO_3_)_2_∙6H_2_O (Panreac, Darmstadt, Germany, 98.0%), were used as metal precursors. In brief, appropriate amounts of the nitrate salts were diluted in distilled water and added to a NaOH (VWR Chemicals, Radnor, PA, USA, 98.9%) precipitating agent solution for co-precipitation. The obtained suspension was filtered, washed, dried, de-agglomerated, and finally calcined in air at 1000 °C for 6 h for the final perovskite structure to be obtained. A series of LS_00_M, LS_30_M, LS_50_M and LS_70_M perovskites were produced.

Half of each perovskite was then impregnated under continuous stirring conditions at 75 °C in an appropriate amount of aqueous solution comprising IrCl_3_·H_2_O (Abcr GmbH & Co KG) with 2 mg Ir/mL in order to achieve the corresponding 2 wt% Ir/LS_X_M catalyst series. After the water evaporated, the obtained suspensions were dried at 110 °C for 12 h; then, they were subjected to reduction at 400 °C for 3 h in a 50 mL/min flow of 25% H_2_/He to remove residual chlorine and to avoid the formation of large Ir crystallites [32,33]. The as-prepared LS_X_M and Ir/LS_X_M catalyst series are listed in Table 1.

### 2.2. Catalyst Characterization Methods

The textural and structural characteristics and redox properties of the LS_Χ_M and Ir/LS_Χ_M catalysts were determined using the N_2_ physical adsorption BET–BJH method, powder X-ray diffraction (XRD), isothermal pulse H_2_-chemisorption (H_2_-Chem.), and Temperature programmed reduction using hydrogen (H_2_-TPR). 

More specifically, the Brunauer–Emmett-Teller (BET) method and Barrett–Joyner–Halenda (BJH) model were used to analyze N_2_ adsorption–desorption isotherms obtained at −196 °C, and relative pressures in the range of 0.05–0.3, using a Quantachrome Nova 2200 e instrument. Subsequently, 150 mg of the sample placed in the instrument holder was degassed for 12 h using a vacuum at 350 °C, prior to measurements. The total specific surface area (S_BET_), the pore volume, and the average pore size diameter of the materials were obtained.

The powder X-ray diffraction (PXRD) patterns of pre-oxidized samples (calcinated in the air at 400 °C for 1 h before the XRD measurements) were collected with a BrukerAXS D8 Advance diffractometer operating at 35 kV and 35 mA, using Cu Kα radiation and a LynxEye detector with a Ni-filter, to determine the crystalline structure of the materials. Measurements were carried out in a 2θ angle range of 4–70 degrees, with a scanning speed of 0.5 degrees per min. The identification and quantification of the phases were performed using BrukerAXS Topas software (COD, Crystallography Open Database) and the Rietveld method.

Pulse hydrogen chemisorption (H_2_-chemisorption) experiments were performed at 25 °C on a Quantachrome/ChemBet Pulsar TPR/TPD instrument equipped with an Omnistar/Pfeiffer Vacuum mass spectrometer to determine iridium dispersion and mean iridium particle size. An amount of 150 mg of the material was loaded on the instrument holder, which was pretreated with a 5% H_2_/He mixture (15 mL/min) at 550 °C for 1 h. Then, it was flushed with N_2_ (15 mL/min) at the same temperature for 30 min, and cooled to 25 °C using a N_2_ flow, before consecutive pulse injections of pure hydrogen (280 µL H_2_ per pulse) were imposed until saturation was reached. This was carried out in order to measure the total H_2_-uptake (V_chem_). The V_chem_ values were then used to calculate iridium dispersion, D_Ir_ (dimensionless, H/Ir ratio), and mean Ir crystallite size, d_Ir_ (in nm) via the following set of Equations (1) and (2) [15,30]:

(1) $$D_{Ir}(H/Ir)=\frac{V_{Chem}\cdot F_{s}{\cdot A}_{Ir}}{V_{mol}\cdot X_{Ir}}$$

(2) $$d_{Ir}(nm)=\frac{6{\cdot A}_{Ir}\cdot 10^{20}}{D_{Ir}\cdot \rho_{Ir}\cdot \alpha_{Ir}\cdot N_{AV}}$$

where, V_Chem_ (mL/g) is the H_2_-uptake in the chemisorption experiment, F_s_ is the hydrogen to metal correlation factor (=2 assuming the one-to-one correlation of adsorbed H atoms with metal sites (i.e., H–Ir)), A_Ir_ is the atomic weight of iridium (192.22 g/mol), V_mol_ is the molar volume of an ideal gas at room temperature and 1 atm pressure (ca. 24,450 mL/mol), X_Ir_ is the iridium content of the catalyst (g_Ir_/g_cat_), ρ_Ir_ is the Ir metal density (22.5 g/mL), α_Ir_ is the cross-sectioned area of the Ir atom (0.12 nm^2^/atom), N_AV_ = 6.023 × 10^23^ molecules/mol is the Avogadro number, and 10^20^ is a unit conversion factor when the units of the parameters in Equations (1) and (2) are used as indicated above.

Temperature-programmed reduction (H_2_-TPR) was performed using the same instrumentation for H_2_-chemisorption experiments in order to obtain the reducibility characteristics and determine the total oxygen storage capacity (t-OSC) of the materials. The samples (150 mg) were oxidized in situ at 750 °C for 30 min (20% O_2_/He flow), then, they were cooled to 25 °C using the same flow, purged for 10 min with He flow. Next, the TPR experiment was performed with a linear (10 °C/min) increase in temperature, up to 750 °C, using 15 mL/min of 1% H_2_/He flow. The time integral of the H_2_-TPR profile determines the total oxygen storage capacity (t-OSC) of the material [13,15,30].

### 2.3. Catalytic Activity and Stability Evaluation Experiments

The catalytic activity and thermal stability experiments were performed in a continuous flow experimental apparatus (Figure S1) consisting of the following: (i) a feed unit utilizing MKS-247 mass flow meters, (ii) a reactor unit with a tubular fixed-bed type reactor (quartz, ID = 3 mm), and (iii) an analysis unit equipped with online gas chromatography (SHIMADZU GC-14B, a thermal conductivity detector, He carrier gas, Porapak-N, and Molecular Sieve 5A columns connected in parallel). The reactor was loaded with m_cat_ = 50 mg of a catalyst in the form of a powder (particle size 180–250 μm). A K-thermocouple, centered in the catalyst bed, was used to measure the reaction temperature. The volume of the catalytic bed was ca. 0.03 cm^3^.

Catalysts were comparatively evaluated for the CH_4_ combustion reaction under conditions of excess O_2_ (1% CH_4_ + 5% O_2_, balance He at 1 bar) at a weight-basis Gas Hourly Space Velocity (WGHSV) equal to 90,000 mL/g∙h (total flowrate F_T_ = 75 mL/min), in the temperature range of 400–970 °C; this corresponds with the operating temperature window of vehicle emission control systems (in particular, of close-coupled catalytic converters). The residence time of the reactants is ca. τ = 0.023 s (i.e., a gas hourly space velocity, GHSV, of ca. 156,500 h^−1^). The ‘light-off’ behavior of the catalysts (CH_4_ conversion versus temperature in constant reactor feed conditions) was obtained by increasing the temperature stepwise (~30 °C/step); in other words, they remained at each temperature for ~20 min to ensure a steady-state operation.

**Scheme 1:**
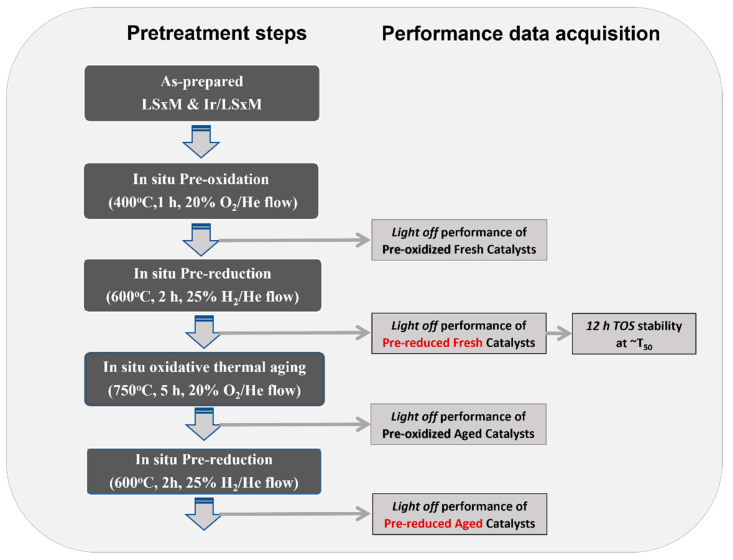
Experimental procedure followed for LS_X_M and Ir/LS_X_M catalysts efficiency and stability evaluation under the complete oxidation of CH_4_ reaction. The evaluation was always performed under reaction conditions comprising 1.0% CH_4_ + 5.0% O_2_ + 94% He, *p* = 1 bar, and WGHSV = 90,000 cm^3^/g∙h (F_T_ = 75 cm^3^/min, m_cat_ = 50 mg).

Both the pre-reduced and pre-oxidized states of the catalyst were evaluated by respectively applying the following pretreatment conditions, prior to light-off measurements: (i) pre-reduction at 600 °C for 2 h with a 50 mL/min flow of 25% H_2_/He, and (ii) pre-oxidation at 400 °C for 1 h with a 50 mL/min flow of 20% O_2_/He. In addition, 12 h of time-on-stream (TOS) stability experiments were performed at a constant temperature, which was different for each catalyst, and equal to that of its corresponding T_50_ (temperature for 50% CH_4_ conversion). Finally, resistance against the deactivation of the LS_Χ_M and Ir/LS_Χ_M counterpart catalysts, after the imposition of stressed oxidative thermal aging conditions, was studied by imposing 5 h in situ oxidation at 750 °C with a 20% O_2_/He flow; then, a re-evaluation of catalyst performances under the same reaction conditions that were previously applied took place. All the above steps, and the order in which they were performed, are shown in Scheme 1.

The conversion of CH_4_ (*X_CH_*_4_) is calculated using Equation (3), as follows:

(3) $$X_{CH_{4}}\left(\%\right)=100\frac{F_{in}{\left[CH_{4}\right]}_{in}-F_{out}{\left[CH_{4}\right]}_{out}}{F_{in}{\left[CH_{4}\right]}_{in}}$$

where F is the total gas flow rate (mL/min), [*CH*_4_] is the *v*/*v* fraction of CH_4_ in the gas stream, and the subscripts “in” and “out” indicate the reactor inlet and outlet gas streams, respectively.

## 3. Results and Discussion

### 3.1. Catalyst Characteristics and Physicochemical Properties

A thorough characterization of the LS_X_M and Ir/LS_X_M materials has been previously performed and can be found in [30]. The textural, morphological, and total oxygen storage capacity (t-OSC) characteristics are summarized in Table 1 and described in detail below.

The specific surface area (S_BET_) values of the materials were between 6.8–12 m^2^/g, which are quite low, but typical for perovskite-type materials. The substitution of La with Sr, up to when X = 50%, decreased S_BET_ monotonically from 12 to 6.8 m^2^/g, which then increased again for when X = 70%, as it approached the initial value. Perovskites are considered to be ionic materials consisting of closed-packed arrays of relatively large oxygen ions. Under these conditions the rate controlling step for densification during sintering comprises the diffusion of the oxygen anions; this takes place through oxygen vacancies rather than through interstitial sites. The addition of the substituted Sr, was compensated with Mn^4+^ cations, the reduction of which, upon heating at 1000 °C, creates oxygen vacancies. These enhance the oxygen anion diffusion rate, and consequently, the densification rate. This explains the gradual S_BET_ reduction, along with the increasing Sr content. Τhe subsequent increase in S_BET_, at higher Sr contents, indicates that at Sr contents higher than 0.5, the defect chemistry of the system changes and is not dictated by the same rules. In addition, the presence of secondary phases, even at small amounts, is able to significantly influence densification during sintering at 1000 °C.

The addition of Ir via impregnation reduced the value of S_BET_ due to the partial blocking of the LS_X_M small-sized pores, caused by the Ir nanoparticles. This is evidenced by the slightly higher average pore size values of Ir/LS_X_M compared with their LS_X_M counterparts (Table 1).

The average size of Ir nanoparticles was estimated to be in the order of 1.0–1.2 nm (Table 1) using Equations (1) and (2) fitted with the H_2_-uptake values obtained from the isothermal H_2_-chemisorption experiments. The corresponding Ir dispersion values (D_Ir_) ranged between 61% and 73%.

The structural characteristics of the materials obtained via the analysis of their XRD patterns confirmed the development of the lanthanum manganate perovskite structure at an angle of 2θ~32.4–33.1° (Figure S2 in the Supplementary Materials file). Other secondary phases of single or mixed oxides, appearing mainly in the material with the higher substitutions of La with Sr (X = 70%), can be seen in Figure S2. The main peak of the perovskite structure, at about 33°, is magnified in Figure S3, wherein a gradual transition from a rhombohedral to a cubic structure (with increasing Sr content) is clearly distinguishable from the splitting of the peak at a diffraction angle 2θ~32–33°. In addition, there is a shift to larger angles as the substitution of La with Sr increases; this indicates that the unit cell is being contracted [47,48]. This cell contraction most likely resulted from the oxidation of Mn^3+^ to Mn^4+^ when the Sr content of the material increased, rather than from the creation of oxygen vacancies, which, if they occurred, would lead to unit cell expansion [47,48]. Moreover, the appearance of other crystalline phases of single or mixed oxides, along with the perovskite structure, appeared in materials with higher substitutions of La with Sr (x = 70%). On the other hand, no Ir species were detected in the XRD diffractograms of Ir/LS_Χ_M catalysts, which is most likely due to its small-sized nanoparticles (~1 nm); this indicates successful dispersion.

**Figure 1:**
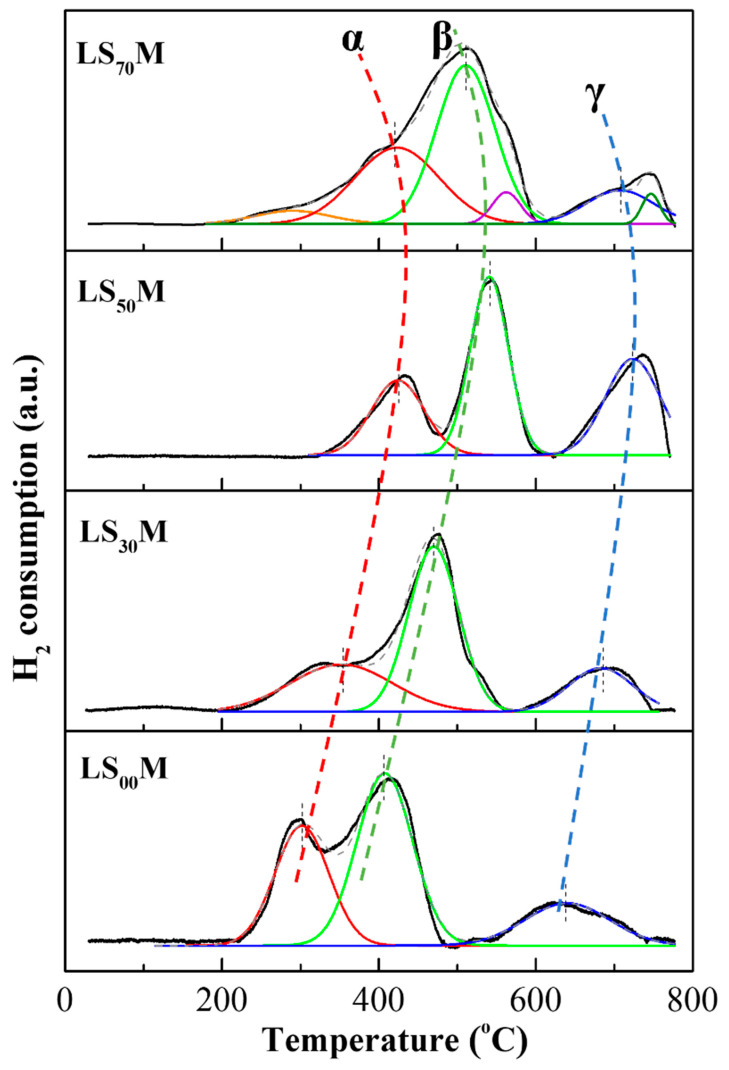
H_2_-TPR profiles for the LS_X_M catalysts and main peak deconvolutions. Peak (α) → O_ads_; peak (β) → Mn^4+^ to Mn^3+^ reduction; peak (γ) → Mn^3+^ to Mn^2+^ reduction.

The reducibility characteristics of the LS_X_M perovskites are shown in the H_2_-TPR profiles of Figure 1. The total oxygen storage capacity (t-OSC) of each perovskite, obtained from the integrated area of their respective TPR profiles in the time interval of the experiment [13,30], are given in Table 1. Values range from 670 to 1350 μmol O_2_/g, increasing systematically with increasing levels of X.

The deconvolution of the broad overlapping peaks in the TPR profiles of LS_X_M reveals three main peaks (α, β, and γ) for the perovskites with compositions where Χ = 0–50%, whereas additional, lower intensity peaks appear when X = 70%. The three main α, β, and γ kinds of oxygen are placed at temperature regions of ca. 200–450 °C, 350–600 °C, and 600–800 °C, respectively; their reduction regions gradually shift to higher temperatures by increasing X until X = 50%, then, they shift again to lower temperatures when X = 70%, in accordance with Ponce et al. [78].

In accordance with the literature, peak α is attributed to adsorbed surface oxygen species, O_ads_; peak β is attributed to the labile lattice oxygen, thus reflecting the Mn^4+^/Mn^3+^ redox couple (Equation (4)) caused by the reduction of high-valence Mn^4+^ to Mn^3+^; and finally, peak γ is attributed to the labile lattice oxygen reflecting the Mn^3+^/Mn^2+^ redox couple (Equation (5)) caused by the reduction of Mn^3+^ to Mn^2+^ [25,44,78,79,80,81,82,83]. Equations (2) and (3) are written in accordance with the Kröger–Vink notation, as follows [84]:

(4) $$O_{O}^{\times }+2{Mn}_{Mn}^{⦁}\overset{{Mn}^{4+}/{Mn}^{3+}}{\Leftrightarrow }2{Mn}_{Mn}^{\times }+V_{O}^{⦁⦁}+\frac{1}{2}O_{2}(g)$$

(5) $$O_{O}^{\times }+2{Mn}_{Mn}^{\times }\overset{{Mn}^{3+}/{Mn}^{2+}}{\Leftrightarrow }2{Mn}_{Mn}^{\prime }+V_{O}^{⦁⦁}+\frac{1}{2}O_{2}(g)$$

where the reference valence for manganese is considered to be +3.

As we shall see below, LS_X_M perovskites are active during lean CH_4_ combustion in the entire temperature region of ca. 250–800 °C, indicating that depending on the operation temperature, all these oxygen types could potentially contribute to the overall methane consumption rate.

Considering the combined XRD and H_2_-TPR findings, it may be concluded that the higher the X, the higher the M^4+^ state content in pre-oxidized LS_X_M, and consequently, the higher the OSC of the material (Table 1). On the other hand, the tendency to reduce manganese (i.e., the lability of lattice oxygen, peaks β and γ) becomes progressively more difficult with increasing levels of X, up to 50%; then, for X = 70%, this is reversed (Figure 1). This can be understood if one considers that gradually increasing quantities of Mn^4+^ are needed to compensate for the Sr^2+^ additions; this finding is in accordance with the results reported in the literature [78], and it is reflected by the fact that the equilibrium constant of the reaction (4) is reported to decrease with increasing levels of X [85].

The addition of Ir to LS_X_M shifts the reducibility peaks of the obtained Ir/LS_X_M material to temperatures as low as ca. 150–400 °C, in which narrow temperature range encompassing all peaks (reflecting Ir^4+^ → Ir^0^, Mn^4+^ → Mn^3+^ and Mn^3+^ → Mn^2+^ reductions) they substantially overlap (Figure S4). This noble metal-induced enhancement of the support’s reducibility is due to an increased spillover of hydrogen atoms from the noble metal particles to the reducible support, thus promoting the reducibility of the latter [74].

The additional, low-intensity peaks that appeared at temperatures of ca. 280, 560, and 750 °C on the LS_70_M H_2_-TPR spectrum presumably originate from the reduction of the additional oxide phases that appear to be present in the XRD pattern of this perovskite (Figure S2). For example, manganate oxide phases can present with reduction peaks at low and intermediate temperature regions following successive reduction processes (MnO_2_ → Mn_2_O_3_ → Mn_3_O_4_ → MnO) [86]. However, the low amount of these phases detected on LS_70_M only marginally affects the overall qualitative/quantitative reducibility behavior of the material, which is still mainly determined by the α, β, and γ peaks (Figure 1).

### 3.2. Light-Off/Light-Out Performance of LS_X_M and Ir/LS_X_M Catalysts

Following the experimental schedule described in Scheme 1 for catalytic performance data acquisition, the light-off (heating)/light-out (cooling) temperature cycles were conducted after different pre-treatment stages, involving fresh or aged (at 750 °C), pre-oxidized or pre-reduced, LS_X_M and Ir/LS_X_M catalysts, respectively. The results of the catalytic behavior, in the order they were obtained (Scheme 1), are discussed below (i.e., first those concerning fresh catalysts are discussed, followed by those concerning thermally aged catalysts, and so on).

#### 3.2.1. Pre-Oxidized Fresh LS_X_M and Ir/LS_X_M Catalysts

**Figure 2:**
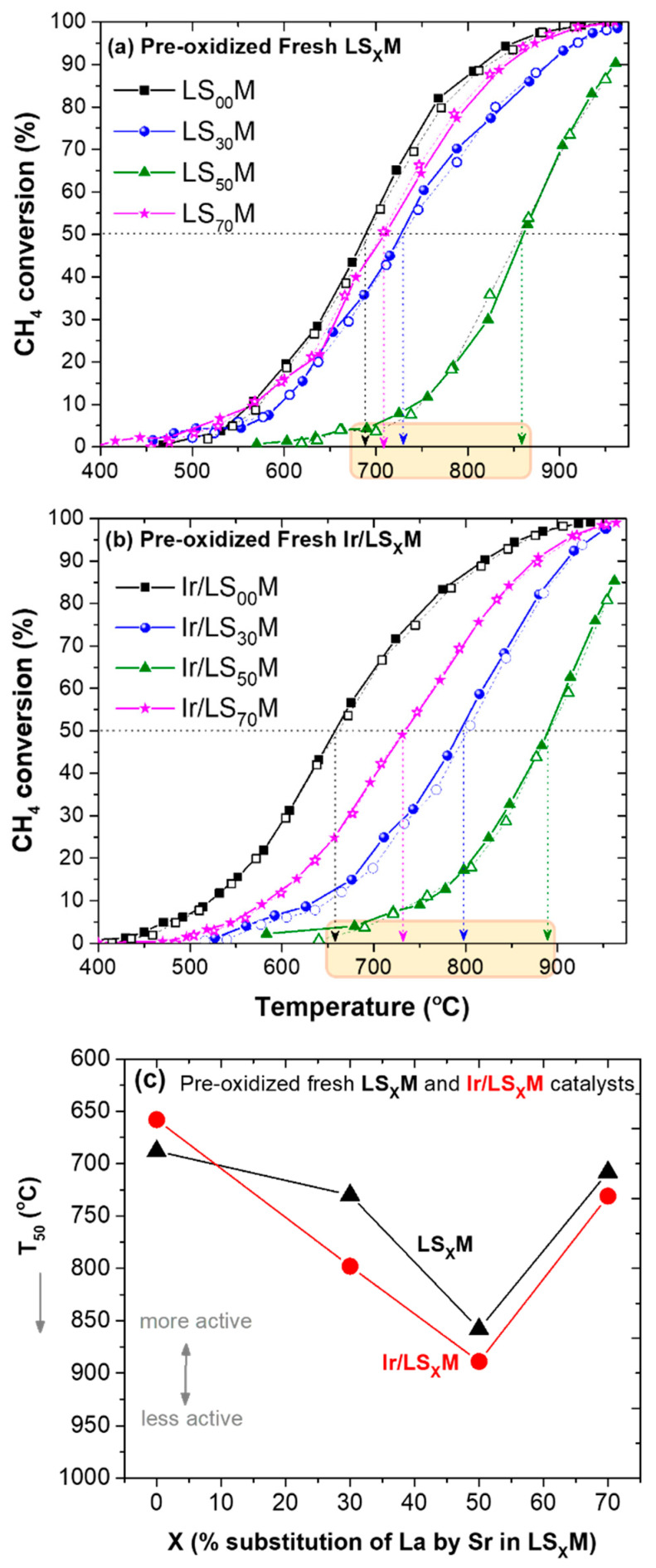
Light-off/light-out behavior of pre-oxidized fresh LS_X_M (**a**) and Ir/LS_X_M (**b**) catalysts, and the corresponding T_50_ values as a function of X (**c**). Feed conditions: 1.0% CH_4_ + 5.0% O_2_, He balance at 1 atm, F_T_ = 75 mL/min, m_cat_ = 50 mg, WGHSV = 90,000 mL/g∙h. Solid symbols and lines in (**a**,**b**) show the heating path, and the open symbols and dashed lines show the cooling path of the cycle.

Figure 2 shows the performance of fresh pre-oxidized LS_X_M (Figure 2a) and Ir/LS_X_M (Figure 2b) catalysts during a heating/cooling cycle. The corresponding T_50_ values (temperature for 50% conversion of CH_4_) are presented in Figure 2c.

It is apparent that the light-off (heating) and light-out (cooling) performances of the CH_4_ conversion coincide to a reasonable extent. Therefore, no hysteresis phenomena were observed. However, the activity of the catalysts appears to be strongly dependent on the La substitution with Sr (X) in the perovskite, which can be seen more clearly with the variation in T_50_ versus Χ (Figure 2c). This dependence, which is qualitatively similar for the two series of materials (LS_X_M and Ir/LS_X_M), is not monotonic, showing inverted volcanic behavior (with respect to activity), with the LS_50_M and Ir/LS_50_M pair appearing to be the less active. Furthermore, and rather unexpectedly, the iridium-loaded perovskites (Ir/LS_X_M) appear typically less active than their pristine perovskite counterparts (LS_X_M).

#### 3.2.2. Pre-Reduced Fresh LS_X_M and Ir/LS_X_M Catalysts

**Figure 3:**
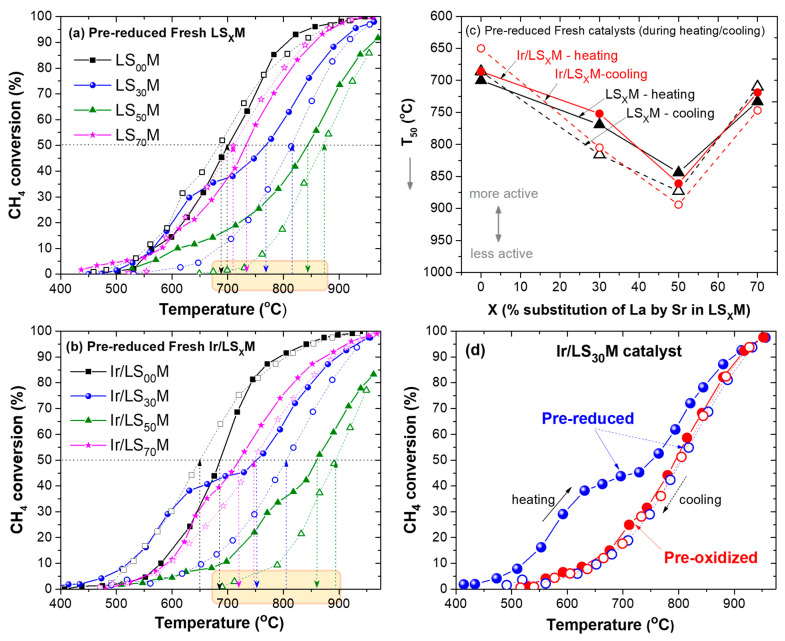
Light-off/light-out behavior of pre-reduced, fresh, LS_X_M (**a**) and Ir/LS_X_M (**b**) catalysts and the corresponding T_50_ values as a function of X (**c**). A selected comparison of the heating/cooling cycles obtained from the pre-reduced (blue symbols and lines) and pre-oxidized (red symbols and lines) Ir/LS_30_M catalysts (**d**). Feed conditions, as shown in Figure 2. Solid symbols and lines show the heating path of the cycles, open symbols and dashed lines show the cooling path of the cycles.

The light-off (heating)/light-out (cooling) behaviors for the two sets of fresh catalysts, LS_X_M and Ir/LS_X_M, starting from their pre-reduced state, are depicted in Figure 3. After this pretreatment, the behavior of the catalysts appears more complex than their pre-oxidized counterparts, as it involved hysteresis phenomena (steady-state rate multiplicity) [30,87,88,89,90]. Both kinds of hysteresis were obtained for the pre-reduced samples during heating/cooling cycles (i.e., normal (counterclockwise) or inverse (clockwise) hysteresis as commonly described in the literature [87], although the latter is clearer (more intense)). In particular, the pair (LS_00_M, Ir/LS_00_M), when X = 0%, follows normal hysteresis (with the activity possessing higher values upon cooling), whereas those with intermediate values, regarding the Sr substitution (X = 30 and 50%), obey inverse hysteresis. The hysteresis behavior of the pair (LS_70_M, Ir/LS_70_M), with the maximum (X = 70%) substitution of La with Sr, is complicated; the former catalyst follows normal hysteresis, and the latter follows inverse hysteresis, although, the amplitude of the hysteresis loop in both cases is limited. Interestingly, the hysteresis loops between the counterpart catalysts, LS_X_M and Ir/LS_X_M, are mirrored with respect to their qualitative characteristics, suggesting that hysteresis mainly originates from the perovskite compartment of the catalyst, rather than from Ir. The latter mainly affects the hysteresis amplitude.

Regarding the activity ranking of the pre-reduced catalysts, this can be followed best using their T_50_ behavior, as depicted in Figure 3c. Similar to that observed for the pre-oxidized samples (Figure 2c), an inverted volcano-type behavior of the activity with increasing substitutions (X) of La with Sr is once again clear (Figure 3c). Thus, LS_X_M and Ir/LS_X_M, when X = 0%, outperformed those when X = 30%, to a greater extent than those when X = 50% (the least active). Then, catalysts with X = 70% appeared more active than those with intermediate X values, approaching the optimal activity of those when X = 0%. Moreover, during the heating part of the light-off/light-out cycle, Ir/LS_X_M catalysts appeared to exhibit a slightly superior performance compared with their LS_X_M counterparts, except in the case of the LS_50_M—Ir/LS_50_M pair (Figure 3c); these behaviors are almost opposite to those recorded for the pre-oxidized samples (Figure 2c). The same is true for the catalysts where X = 0 and 30% (low Sr content), but not for cases where X = 50% and 70% (high Sr content) during the cooling part of the cycle.

When comparing the behaviors of pre-reduced and pre-oxidized LSxM (Figure S5a) and Ir/LSXM (Figure S5b) catalysts, a clear trend is revealed; in the vast majority of cases, the cooling part of the pre-reduced catalysts better approximates, or even coincides with, the behavior of their pre-oxidized counterparts. A typical example (for the Ir/LS_30_M catalyst) is shown in Figure 3d.

### 3.3. Light-Off/Light-Out Performance of LS_X_M and Ir/LS_X_M Catalysts Aged at 750 °C

Herein, the behavior of CH_4_ conversion as a function of temperature during heating/cooling cycles between 400 and 900 °C is presented for LS_X_M and Ir/LS_X_M catalysts that were previously subjected to oxidative thermal aging for 5 h, as described in Scheme 1. Both the pre-oxidized and pre-reduced states of the samples at the beginning of the cycle were investigated.

#### 3.3.1. Pre-Oxidized Catalysts Aged at 750 °C

**Figure 4:**
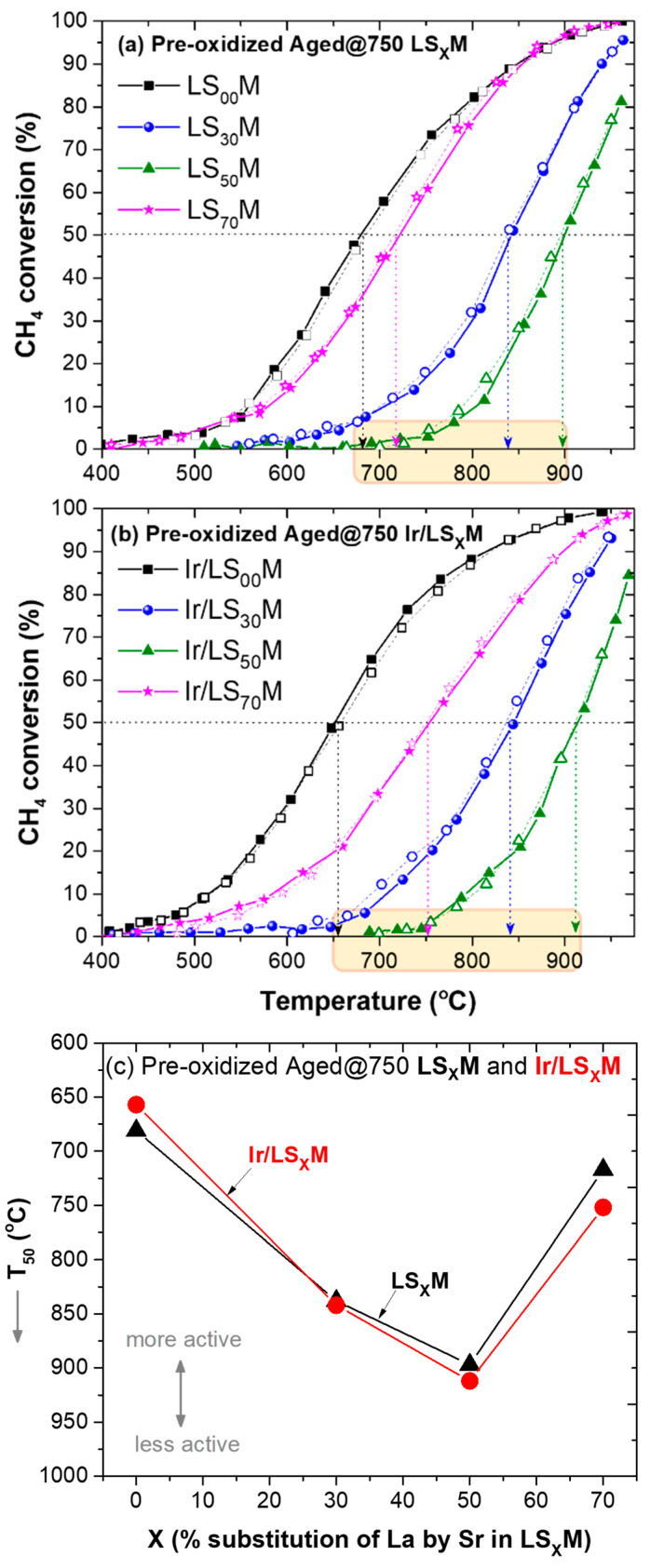
Light-off/light-out behavior of pre-oxidized, aged at 750 °C, LS_X_M (**a**) and Ir/LS_X_M (**b**) catalysts, and their corresponding T_50_ values as a function of X (**c**). Feed conditions are shown, as in Figure 2. Solid symbols and lines in (**a**,**b**) show the heating path and open symbols, and the dashed lines show the cooling path of the cycle.

Figure 4 presents the CH_4_ conversion results obtained during the heating/cooling cycle for the pre-oxidized LS_X_M (Figure 4a) and the Ir/LS_X_M (Figure 4b) catalysts that were aged at 750°C, whereas Figure 4c depicts the corresponding T_50_ behavior of the catalysts as a function of X. As for fresh catalysts (Figure 2), no hysteresis phenomena were observed during the cycles over the pre-oxidized, aged at 750°C samples. It was also observed that pure perovskites (LS_X_M) slightly outperformed their Ir-doped counterparts (Ir/LS_X_M), except for LS_00_M (Figure 4c).

Regarding the dependence of the activity on the Sr content of LS_X_M, the aged catalysts maintained the same behavior for their fresh counterparts (Figure 2). It is once again an inverted volcanic-type dependent activity competing against X, with LS_50_M and Ir/LS_50_M catalysts exhibiting inferior performances, with T_50_ values approximately 250 °C higher than those for LS_00_M and Ir/LS_00_M (Figure 4c).

#### 3.3.2. Pre-Reduced Catalysts Aged at 750 °C

**Figure 5:**
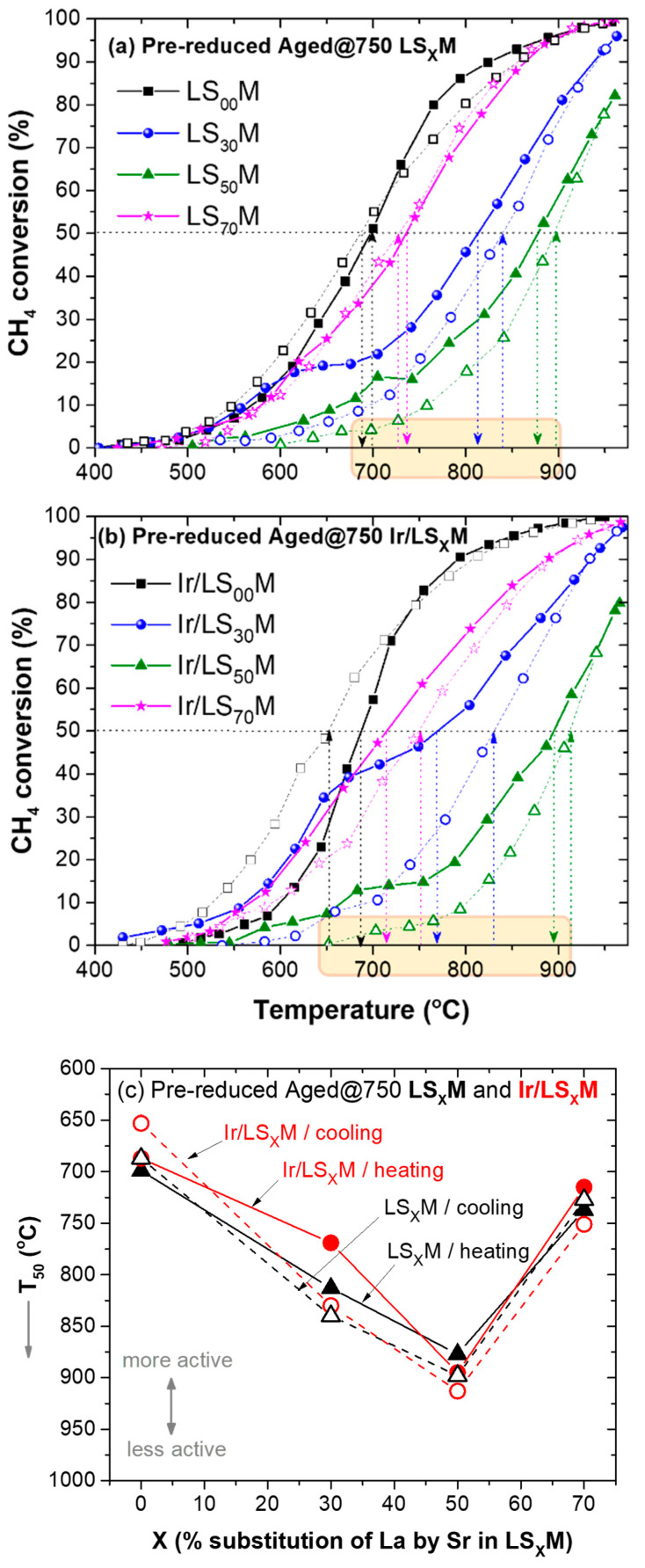
Light-off/light-out behavior of pre-reduced, aged at 750 °C, LS_X_M (**a**) and Ir/LS_X_M (**b**) catalysts, and their corresponding T_50_ values as a function of X (**c**). Feed conditions are shown, as in Figure 2, Figure 3 and Figure 4. Solid symbols and lines show the heating path, and open symbols and dashed lines show the cooling path of the cycle.

The performance of the pre-reduced LS_X_M and Ir/LS_X_M catalysts that were aged at 750 °C, and their impact on the lean CH_4_ oxidation reaction during heating/cooling cycles, are depicted in Figure 5a,b, respectively. There are no significant differences compared with what was already observed in the behavior of their fresh counterparts. Again, hysteresis phenomena, which are more pronounced for the intermediate values of the percentages where La was substituted for Sr (X = 30 and 50%), were observed.

The CH_4_ conversion efficiency performance versus X of the pre-reduced catalysts aged at 750 °C, in terms of T_50_, is shown in Figure 5c. There are some variations in T_50_ values between the heating and cooling parts of the cycle due to the hysteresis, which generally indicates more active catalysts in the heating part of the loop. Since the thermal cycle starts with pre-reduced catalysts, it is possible that in this part of the cycle, the pre-imposed metallic Ir^0^ is partially oxidized by the oxidizing environment of the reactants (lean conditions), creating an IrO_2_/Ir^0^ complex state. As demonstrated by Rui and co-workers [91], this offers favorable sites for the overall methane oxidation reaction, compared with metallic Ir^0^ or stoichiometric IrO_2_. In the same vein as the results of Schick et al. [31], regarding the total oxidation of VOCs over Ir/SiO_2_ catalysts, which showed that surface Ir^3+^ species are associated with improved catalyst performance as well as size–activity relationship; regarding the latter, this is because as the size of the Ir nanoparticles decreases, the defective Ir^3+^ active sites increase.

However, when the system achieves 100% CH_4_ conversion at a high temperature, i.e., it operates in net oxidizing conditions, (due to the remained unconsumed O_2_, the Ir nanoparticles are fully oxidized into stoichiometric IrO_2_, therefore, they become less active in the reaction, and during its return to low temperatures (cooling) the system exhibits lower activity.

Nevertheless, the effect of substituting La with Sr (X) remains the main cause of strong changes in terms of the efficiency of the catalysts. Changes in the order of ~60 °C max. were obtained between the heating and cooling sections of the hysteresis loop; these are insignificant compared with changes of 250 °C, caused by the 50% replacement of La with Sr.

### 3.4. Thermal Aging and Time-on-Stream Stability of Catalysts

**Figure 6:**
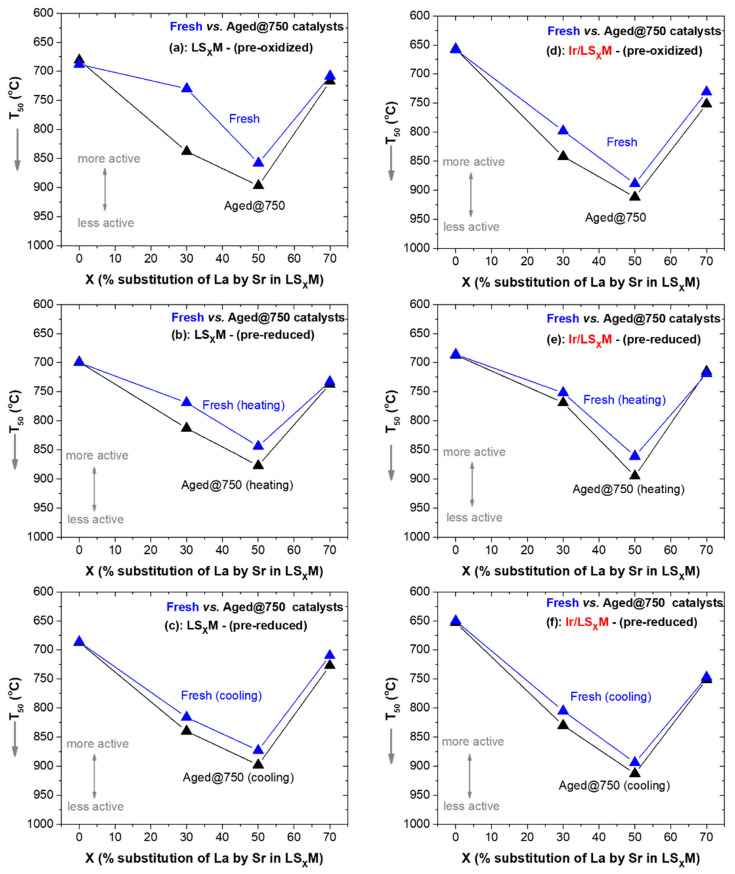
Comparison of fresh and aged at 750 °C LS_X_M and Ir/LS_X_M catalysts in T_50_ vs. X diagrams. (**a**–**c**) correspond with LS_X_M; (**d**–**f**) correspond with Ir/LS_X_M. (**a**,**d**) compare fresh vs. aged (at 750 °C) pre-oxidized LS_X_M and Ir/LS_X_M catalysts, respectively; (**b**,**c**) compare fresh vs. aged (at 750 °C) pre-reduced LS_X_M and Ir/LS_X_M catalysts during heating, respectively; (**c**,**f**) compare fresh vs. aged (at 750 °C) pre-reduced LS_X_M and Ir/LS_X_M catalysts during cooling, respectively. Feed conditions are shown, as in Figure 2, Figure 3, Figure 4 and Figure 5.

In order to evaluate the thermal stability of the materials after prolonged exposure to thermal aging at high temperatures under oxidizing conditions (5 h at 750 °C in a 20% O_2_/He flow), Figure 6 was constructed, which presents the efficiency (in terms of T_50_) of fresh and aged (at 750 °C) LS_X_M (Figure 6a–c) and Ir/LS_X_M (Figure 6d–f) catalyst series.

For the pre-oxidized catalysts of each series, LS_X_M and Ir/LS_X_M, which do not show hysteresis after such a pretreatment, one diagram is sufficient for their comparison (Figure 6a for LS_X_M and Figure 6d for Ir/LS_X_M). However, for pre-reduced catalysts exhibiting hysteresis, a comparison is made with respect to both the heating and cooling parts of the hysteresis loops (Figure 6b,c for LS_X_M and Figure 6e,f for Ir/LS_X_M).

It is evident from Figure 6 that aging causes the degradation of the catalytic efficiency of the studied materials, but this is rather insignificant, as in most cases, this results in only slight increases in T_50_ (ca. +20 °C). Furthermore, in the case of catalysts with X = 0 and 70%, the activity of the catalysts, either of unloaded or Ir-loaded perovskites, remains unaffected after aging. The thermal aging stability of perovskites under oxidizing conditions is well established, and its confirmation it was not surprising to obtain this result for the pristine LSxM (Figure 6a–c). Moreover, the strong susceptibility and tendency to aggregate, regarding the supported iridium nanoparticles under oxidative thermal aging conditions, is also known [37,38], and thus, it was the focus of the stability experiments. However, the results of Figure 6d–f are similar to those of the pristine perovskites; the Ir/LS_X_M catalysts exhibited good stability, and an explanation for this is given below.

**Figure 7:**
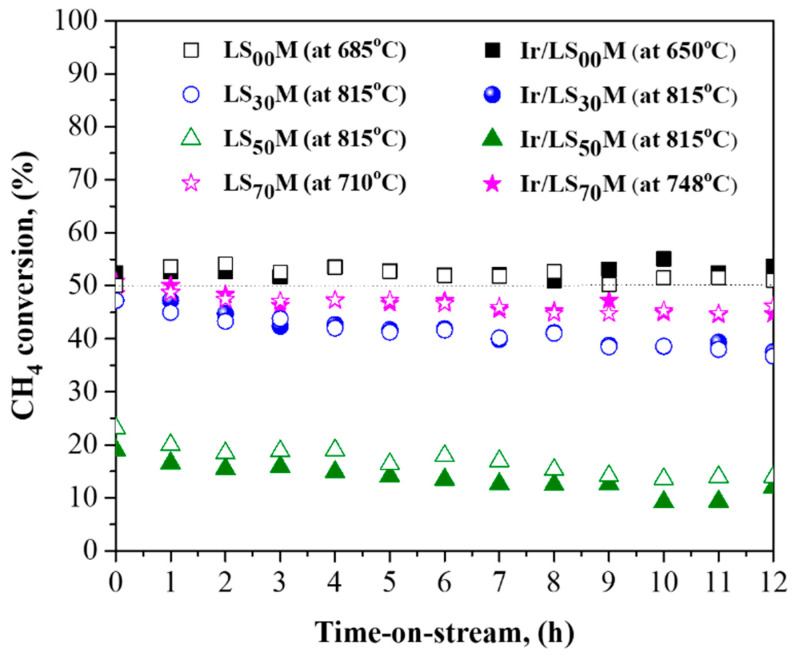
A 12 h time-on-stream stability test for the LS_X_M (open symbols) and Ir/LS_X_M (closed symbols) catalysts. Conditions: 1.0% CH_4_ + 5.0% O_2_, He balance at 1 atm, F_T_ = 75 mL/min, m_cat_ = 50 mg, WGHSV = 90,000 mL/g∙h. Regarding the temperature (indicated in figure inset), it was close to T_50_ for each catalyst, except for the less active pair, LS_50_M and Ir/LS_50_M.

Regarding the time-on-stream stability (TOS) of our catalysts, experiments were performed for a period of 12 h (Figure 7) under constant temperatures that corresponded with the T_50_ exhibited by each of them. The less active catalysts, LS_50_M and Ir/LS_50_M, with high T_50_ values (~900 °C), were operated at temperatures of 850 °C; at such temperatures, they have an initial CH_4_ conversion rate of about 20–25% (Figure 7). All catalysts showed very good stability, since only a slight degradation, in the order of 5%, was recorded after 12 h of operation. It is worth noting that the catalyst pair, LS_00_M and Ir/LS_00_M, was the most active compared with all others investigated, and it also offered the best TOS stability, with zero degradation in terms of their efficiency, over the 12 h period.

### 3.5. Main Observations and Material Properties—Catalytic Efficiency Correlations

The main observations of the above comparative studies of catalytic behavior (efficiency and stability) of the two series of LS_X_M and Ir/LS_X_M catalysts for the complete CH_4_ oxidation reaction under lean conditions, can be summarized as follows:

(i) The most important determining factor of the materials, for all cases (unloaded or Ir-loaded LS_X_M, pre-oxidized or pre-reduced, and fresh or aged materials), in terms of efficiency, was the composition of the LS_X_M perovskite, specifically, the amount of La that was substituted with Sr (X = 0, 30, 50, and 70%). This key factor typically causes shifts in T_50_ of up to ca. 300 °C, whereas other parameters appeared capable of shifts in T_50_ that were one order of magnitude lower (ca. 30 °C) (Figure 2, Figure 3, Figure 4 and Figure 5).
(ii) X variation produced an inverted volcanic-type effect in terms of catalytic efficiency, with the most active in all cases the catalysts that did not contain Sr, i.e., X = 0% (LS_00_M = LaMnO_3_ and Ir/LS_00_M = Ir/LaMnO_3_), and less active the catalysts with X = 50% (i.e., LS_50_M and Ir/LS_50_M). Catalysts with larger X value (X = 70%; La_0.3_Sr_0.7_MnO_3_ and Ir/La_0.3_Sr_0.7_MnO_3_), tended to be similar to the behavior of the optimal catalysts (LaMnO_3_ and Ir/LaMnO_3_) (Figure 2c, Figure 3c, Figure 4c, Figure 5c and Figure 6).
(iii) The addition of Ir nanoparticles onto the LS_X_M surface did not perform as expected. It had a negative effect on the efficiency of pre-oxidized catalysts (Ir/LS_X_M show T_50_ values that were ~30°C higher than those of their non-loaded LS_X_M counterparts) (Figure 2c and Figure 4c), whereas the pre-reduced catalysts exhibited a small positive effect at high temperatures, but it was more noticeable for lower temperatures (Figure 3c and Figure 5c).
(iv) Hysteresis phenomena during heating/cooling cycles appeared only in the case of pre-reduced catalysts for both LS_X_M and Ir/LS_X_M series (Figure 3 and Figure 5), whereas for pre-oxidized materials, the light-off and light-out curves faithfully coincided with each other (Figure 2 and Figure 4).
(v) The hysteresis loops are qualitatively similar for the corresponding LS_X_M and Ir/LS_X_M catalysts, which indicates that the phenomenon is mainly determined by LS_X_M and its properties, rather than Ir (Figure 3 and Figure 5).
(vi) The oxidative thermal aging of the materials caused marginal decreases in their efficiency (i.e., less than ~40 °C shifts of T_50_ towards higher temperatures). However, for the most active catalysts (with X = 0 and 70%), no activity deterioration was recorded (Figure 6).
(vii) The time-on-stream stability of the materials was generally good, as the efficiency (methane conversion) only declined by 5–10%; this was observed after 12 h of operation. Notably, no degradation in the catalytic efficiency of the optimal LS_00_M (LaMnO_3_) and Ir/LS_00_M (Ir/LaMnO_3_) catalysts was recorded (Figure 7).

**Figure 8:**
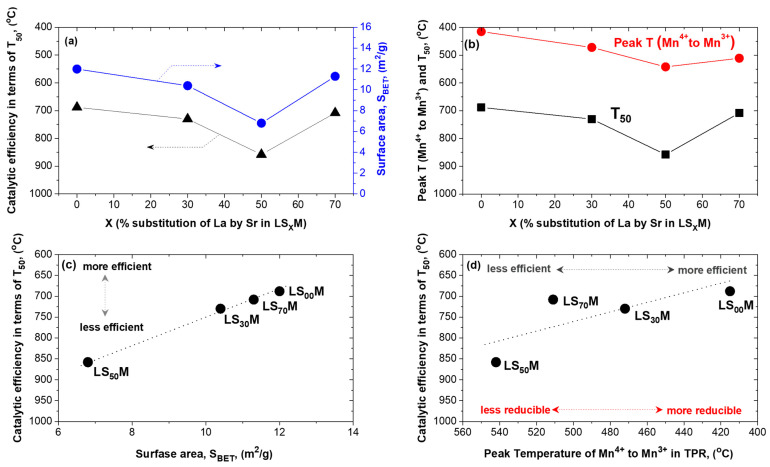
Correlations between the LS_X_M lean CH_4_ combustion efficiency and its textural characteristics and properties, which were mainly changed as a result of the degree of Sr substitution (X). (**a**) Variation in LS_X_M surface area and efficiency (T_50_) as a function of X; (**b**) variation of LS_X_M reducibility (in terms of peak temperature of the Mn^4+^ → Mn^3+^ reduction in the TPR profile) and efficiency (T_50_) as a function of X; (**c**) direct correlation of LS_X_M catalytic efficiency with its surface area; and (**d**) direct correlation of LS_X_M catalytic efficiency with its reducibility.

After a close comparison of the LS_X_M characteristics (and how these change in accordance with the extent (X) to which La was substituted with Sr) with the catalytic results, we can readily conclude that the main factors affecting the activity of the perovskites during the lean CH_4_ combustion are the total surface area (S_BET_) and the reducibility of the perovskite. Indeed, as shown in Figure 8a and b, respectively, the efficiency of the LS_X_M (in terms of T_50_) faithfully mimics the changes in both the total surface area (S_BET_) and reducibility (expressed in terms of the peak temperature of Mn^4+^ → Mn^3+^ reduction in the TPR profile of Figure 1) when the level of substituted Sr (X) in the perovskite increases.

Studies of the literature, regarding lean CH_4_ combustion over perovskite-type materials, typically describe the reaction mechanism via the Eley–Rideal (ER), Mars–van Krevelen (MVK), and/or both (two-term) kinetic models [63,67,79]. The ER model involves the direct reaction of gas phase methane with adsorbed oxygen species (O_ads_) on the perovskite surface. The MVK model implicates the reaction of gas phase CH_4_ with surface lattice oxygen (Equation (6)), the latter being continuously replenished by the dissociative chemisorption of O_2_ on surface oxygen vacancies (Equation (7)), as follows:

(6) $$4O_{O}^{\times }+CH_{4}\to CO_{2}+2H_{2}O$$

(7) $${\frac{1}{2}O}_{2}(g)\overset{}{\Leftrightarrow }O_{O}^{\times }$$

Performing Density Functional Theory (DFT) calculations for CH_4_ combustion on LSM perovskites, Wang et al. [92] also indicated that CH_4_ adsorption and activation (CH_4_* → CH_3_* + H*) occurs on Mn sites. They argued that this activation, revealed to be more facile on the (001) rather than (110) crystallographic plane, can cause further enhancements to catalytic methane oxidation after a reaction concerning such methane-derived species with neighbor lattice oxygen atoms takes place.

It is therefore obvious from the above considerations that perovskite activity, regarding the reaction under consideration, will be directly proportional to the population of labile oxygen species on the perovskite surface, and thus, to the perovskite surface area itself. At the same time, the degree of lability of these lattice oxygen species would act synergistically upon the enhancement of the activity.

Observations (i) and (ii) that are related to the entire catalytic efficiency data, either obtained on fresh or aged, or on pre-oxidized or pre-reduced, catalysts, they exhibit an activity order (LS_00_M ~ LS_70_M > LS_30_M > LS_50_M) that is fully consistent with the above. The higher the perovskite surface area and lattice oxygen lability, the higher its efficiency in terms of lean CH_4_ combustion (Figure 8c,d). As we have seen here, increasing the amount of substituted Sr in LS_X_M, up to X = 50%, gradually degrades both of these activity-determining parameters; for X = 70%, however, the formation of other simple oxide phases (e.g., Mn_2_O_3_, see XRD results in Figure S2) may be the cause of the recovery of high values in said properties, and therefore, the return of efficiency to high values.

The unexpected inhibitory effect of adding Ir to the surface of LS_X_M perovskites, regarding their catalytic efficiency (observation iii), is still uncertain. As shown in Table 1, the Ir/LS_X_M catalysts typically have a lower surface area compared with their LS_X_M counterparts due to their partially blocked pores, as explained in Section 3.1. Therefore, part of the surface of the perovskite support, which is intrinsically active in the reaction, is rendered inaccessible to the reactants mixture. The catalyst thus loses active centers, which, in the case of pre-oxidized samples, are not replenished by the presence of, relatively inactive to the reaction, IrO_2_ phase. In the case of the pre-reduced Ir/LS_X_M catalysts, this undesirable effect of the iridium species is partially compensated with the creation of an active for the reaction IrO_2_/Ir^0^ phase (partially oxidized iridium) [31,91]. This occurs after the exposure of the Ir^0^ species of the pre-reduced catalysts to the excess O_2_ conditions. However, as our results show, this favorable partially oxidized surface of the iridium particles (IrO_2_/Ir^0^) created on the pre-reduced catalysts is not expected to remain in high temperatures and under excess O_2_ reaction conditions. It transforms to the less active IrO_2_ which moderates positive effects (Figure 3 and Figure 5).

The consistent interpretations of observations (iv) and (v), which are related to inverse hysteresis phenomena (Figure 3 and Figure 5), and are recorded in this study, are as follows:

Why is hysteresis observed only on the pre-reduced, and not the pre-oxidized, catalysts, and why is it of the inverse type (i.e., clockwise, the heating part outperforms its cooling part)? The light-off (heating part) behavior of the pre-reduced catalysts (Figure 3 and Figure 5) is very similar to the behavior first observed by Arai et al. [67], who studied CH_4_ combustion on La_0.8_Sr_0.2_MnO_3_ and La_0.6_Sr_0.4_MnO_3_ perovskites using a reaction mixture of 2% *v*/*v* CH_4_ in air. It exhibits a characteristic “hump” in the low-temperature region, then, it changed curvature at higher temperatures, as also found herein (Figure 3 and Figure 5). We agree with the explanation given by the authors, as follows. The initial high rate of CH_4_ consumption is the result of the contribution to the overall rate of adsorbed oxygen species (O_ads_) on the perovskite surface which is then limited by increasing temperatures (at high temperatures the O_ads_ desorb rapidly); however, at high temperatures the participation of lattice oxygen ($O_{O}^{\times }$) in methane oxidation becomes dominant [67].

Regarding the behavior of the cooling (light-out) part of the hysteresis loops (Figure 3 and Figure 5), we can give the following rational explanation. When approaching ~100% methane conversion at the upper-temperature limit, the catalyst experiences pure oxidative conditions (~100% CH_4_ conversion, excess O_2_). It is therefore oxidized under such conditions, and by lowering the temperature, it follows a path of lower activity, similar to the results obtained in pre-oxidized samples (Figure 2 and Figure 4). This explains why the cooling part of the hysteresis loops was always similar to the light-off curves of the pre-oxidized catalysts, in addition to the “inverse” character of the hysteresis.

In accordance with the above, hysteresis mainly occurs due to the favorable contribution of O_ads_ species to the total methane consumption rate. However, the reason why this was found to only work for pre-reduced samples, but not pre-oxidized samples, can be interpreted as follows. The pre-reduction process of LSxM materials is expected to enhance the population of oxygen vacancies on their surface due to a partial Mn^4+^ → Mn^3+^ reduction. When such a pre-treated LS_X_M perovskite experiences in methane oxidation environments, the gas phase dioxygen interacts with oxygen vacancies, providing two atomic oxygens, one of which is bound by the vacancy that becomes the lattice oxygen, and the other can diffuse to the perovskite surface in the form of adsorbed O_ads_. Thus, a mechanism of continuous replacement concerning both lattice and adsorbed oxygen species is at work during the reaction on pre-reduced materials. Conversely, the pre-oxidized perovskites, due to the lack of oxygen vacancies on their surface to activate the dissociative chemisorption of gaseous dioxygen in this temperature range, have a limited amount of O_ads_ that impose low reaction rates. Thus, for per-oxidized samples, both the heating and cooling parts of the light-off experiments coincide since the samples work for both paths in their oxidized state.

The presence of Ir in the catalyst formulations (Ir/LS_X_M) appears to have some noticeable effect on the hysteresis phenomena, especially on loop amplitude, regarding CH_4_ conversion. It appears strongly enlarged (Figure 5b) compared with that recorded in the corresponding LS_X_M catalysts (Figure 5a). A reasonable explanation is that the partially oxidized IrO_2_/Ir^0^ sites (their creation has been described above), which are favorable for the dissociative adsorption of gaseous dioxygen, further facilitate this necessary mechanistic step of the reaction. The as-derived oxygen atoms can then directly react with the methane, and/or spillover to the perovskite surface, thus replenishing the lattice oxygen consumed by the reaction, enhancing methane combustion.

Regarding observation (vi), which concerns the good stability of our materials in spite of the fact that they were subjected to arduous conditions, even those containing Ir (Ir/LS_X_M), is an impressive finding considering that Ir is a very sensitive catalyst with regard to thermal aging; it has a high aggregation tendency, even at temperatures as low as 450°C [13,14,37,38,39,40,41]. The present results once again confirm our recent experimental findings, theory, and developed model, demonstrating that supports possess a sufficient population of bulk and surface O-defects (oxygen vacancies), and a high mobility of lattice O^2−^ ions effectively act against the thermal sintering of catalyst nanoparticles dispersed on their surface [40,41]. The perovskites used herein as supports for Ir nanoparticles meet these requirements.

Observation (vii), on the other hand, concerning the good time-on-stream stability of our materials, most likely reflects the absence of the accumulative carbon deposition phenomena originating from the methane cracking reaction, the occurrence of which is not thermodynamically excluded in the temperature range used for the reaction in this study.

An additional aspect worth noting in the present findings concerns the fact that, in the literature, it is known that at high temperatures (ca. > 850 °C, reached in the present study) the contribution of the gas phase to the total methane oxidation can be appreciable under certain conditions and should not be ignored. Matzaras and co-workers, in a Cartesian plot of SV (surface-to-volume ratio) versus residence time, delineated the regimes concerning the significant involvement of gas-phase chemistry for given pressures and temperatures [93]. Considering the conditions in which the present experiments were performed (SV > 200,000 and residence time ~0.017 s at a pressure of 1 bar), it seems that the catalytic system operates under conditions that starkly differ with those wherein the contribution of the gas-phase reaction could be noticeable. This view is further strengthened by two additional facts, as follows: first, no slope change tendency of the light-off curves at high temperatures is observed in Figure 2, Figure 3, Figure 4 and Figure 5; second, nor was CO detected in the reaction products (the contribution of the gas phase goes through the reaction sequence: CH_4_ → CO gas-phase route followed by the catalytic oxidation of the produced gaseous CO to CO_2_). 

Finally, taking into account the small particle size of the catalytic powdered samples (180–250 μm), and the high GHSV used (ca. 156,000 h^−1^), mass and heat transfer limitations, if any, should be negligible.

## 4. Conclusions

The complete oxidation of the methane reaction was investigated in detail on a series of LS_X_M (X = % substitution of La with Sr = 0, 30, 50, and 70%) perovskites, and on their 2 wt% Ir-loaded counterpart catalysts.

The key parameter affecting efficiency for both series concerned the degree (X) of substitutional Sr, rather than the addition of Ir; the latter caused a slight inhibition of activity, rather than promotion, when in its oxidation state (IrO_2_), and it notably enhanced activity when in a partially oxidized state (IrO_2_/Ir^0^).

The main factors through which parameter Χ affects the activity were the changes it induced in the materials’ total surface area and on their reducibility that act synergistically.

The materials’ efficiency followed an inverted volcanic behavior pattern, as a function of X, with the most efficient catalysts being the LS_00_M—Ir/LS_00_M pair, and the least efficient being the LS_50_M—Ir/LS_50_M pair; this strikingly reflects the effect of X on the total surface area and the reducibility of the materials. Large differences in T_50_ (ca. 300 °C) were recorded between the most and least active catalysts.

The inhibitory effect on efficiency, caused by the addition of Ir to the perovskite, was attributed to partial pore blocking, and consequently, to a reduction in the number of active sites, in combination (for pre-oxidized catalysts) with the low activity of IrO_2_ particles. For pre-reduced catalysts, the creation under reaction conditions of a reactive partially oxidized IrO_2_/Ir^0^ phase enhanced activity, especially at low temperatures.

Inverse hysteresis phenomena recorded on pre-reduced, but not pre-oxidized, LSxM and Ir/LS_X_M catalysts during light-off (heating)/light-out (cooling) temperature cycles and overall reaction kinetics were consistently interpreted by assuming that CH_4_ oxidation was performed in parallel with O_ads_ species (Eley–Rideal model) and lattice oxygens (Mars–van Krevelen model) (i.e., a two-term kinetic model).

Both the LS_X_M and Ir/LS_X_M series of materials were found to be very stable in both oxidative thermal aging tests at 750 °C for 5 h, and the 12 h time-on-stream stability tests under the reaction’s conditions.

The great stability of perovskites at high temperatures for the total oxidation of CH_4_, the convenience they provide for effectively tailoring their performance via the partial substitution of the A or B sites with other cations, and the efficient CH_4_ conversion they achieve, even without noble metal loading, make them promising materials that are worthy for future studies concerning demanding applications such as exhaust gas treatment in NG-driven vehicles or gas turbines (GTs).

## Figures and Tables

**Table 1:** The LS_Χ_M (Χ = % substitution of La with Sr) and 2wt% Ir/LS_Χ_M series of catalysts studied. A summary of their chemical formulas, textural, morphological, and oxygen storage capacity characteristics [30].

Catalysts Code	Chemical Formula	S_BET_ (m^2^/g)	Average Pore Diameter (nm)	Total OSC (μmol O_2_/g)	Mean Ir Particle Size (nm)
LS_00_M	LaMnO_3_	12.0	10.9	671	n.a
LS_30_M	La_0.7_Sr_0.3_MnO_3_	10.4	9.8	766	n.a
LS_50_M	La_0.5_Sr_0.5_MnO_3_	6.8	8.9	886	n.a
LS_70_M	La_0.3_Sr_0.7_MnO_3_	11.3	8.8	1219	n.a
Ir/LS_00_M	2 wt% Ir/LaMnO_3_	9.7	11.9	753	1.1
Ir/LS_30_M	2 wt% Ir/La_0.7_Sr_0.3_MnO_3_	10.5	10.0	981	1.1
Ir/LS_50_M	2 wt% Ir/La_0.5_Sr_0.5_MnO_3_	6.2	8.1	1203	1.0
Ir/LS_70_M	2 wt% Ir/La_0.3_Sr_0.7_MnO_3_	11.0	13.7	1348	1.2

## Data Availability

The data are available from the laboratory of origin upon request www.pccplab.tuc.gr (accessed on 1 March 2023).

## Supplementary Materials

The following supporting information can be downloaded at: https://www.mdpi.com/article/10.3390/nano13152271/s1: Figure S1. Schematic configuration of the continuous flow experimental apparatus equipped with on-line Gas Chromatography; Figure S2. XRD patterns of LS_X_M perovskites (La_1−x_Sr_x_MnO_3_; X = 0, 30, 50 and 70% substitution of La by Sr) and the corresponding 2 wt% Ir/LS_X_M catalysts; Figure S3. Magnification of the XRD patterns of LS_X_M perovskites at the region 2θ = 32–34°, where the main peak of the perovskite phase appears; Figure S4. H_2_ consumption versus temperature (H_2_-TPR profiles) of Ir/LS_X_M catalysts. Figure S5. Dependence of T_50_ on X (% substitution of La by Sr in the perovskite composition) for pre-reduced and pre-oxidized LS_X_M catalysts (a), and pre-reduced and pre-oxidized Ir/LS_X_M catalysts (b); Table S1. Temperature for 50% CH_4_ conversion (T_50_) of pre-oxidized and pre-reduced, fresh LS_X_M and Ir/LS_X_M catalysts; Table S2. Temperature for 50% CH_4_ conversion (T_50_) of pre-reduced and pre-oxidized LSxM and Ir/LSxM catalysts that were aged at 750 °C.
