# Supplementary material for: Methane Catalytic Combustion under Lean Conditions over Pristine and Ir-Loaded La1−xSrxMnO3 Perovskites: Efficiency, Hysteresis, and Time-on-Stream and Thermal Aging Stabilities
Source: Nanomaterials (Basel). 2023 Aug 7;13(15):2271. doi: 10.3390/nano13152271 (PMC10420673; doi:10.3390/nano13152271)
Supplement: Supplementary file 1 [file nanomaterials-13-02271-s001.zip › nanomaterials-2528278-supplementary.pdf]

Supplementary Material

Methane Catalytic Combustion under Lean Conditions over Pristine and Ir-Loaded $La_{1-x}Sr_xMnO_3$ Perovskites: Efficiency, Hysteresis, and Time-On-Stream and Thermal Aging Stabilities

Catherine Drosou [1,*], Ersi Nikolaraki [1], Theodora Georgakopoulou [1], Sotiris Fanourgiakis [1], Vassilios T. Zaspalis [2,3] and Ioannis V. Yentekakis [1,4,*]

[1] Laboratory of Physical Chemistry & Chemical Processes, School of Chemical and Environmental Engineering, Technical University of Crete, 731 00 Chania, Crete, Greece; enikolaraki@tuc.gr (E.N.); theodorageorgakopoulou@gmail.com (T.G.); sfanourgiakis@tuc.gr (S.F.)

[2] Department of Chemical Engineering, Aristotle University of Thessaloniki, 541 24 Thessaloniki, Greece; zaspalis@auth.gr

[3] Chemical Process & Energy Resources Institute, Center for Research & Technology Hellas (CPERI/CERTH), 570 01 Thermi, Thessaloniki, Greece

[4] Institute of GeoEnergy, Foundation for Research and Technology-Hellas (FORTH/IG), 731 00 Chania, Greece

* Correspondence: edrosou@tuc.gr (C.D.); igentekakis@tuc.gr (I.V.Y.)

Supplementary Figures

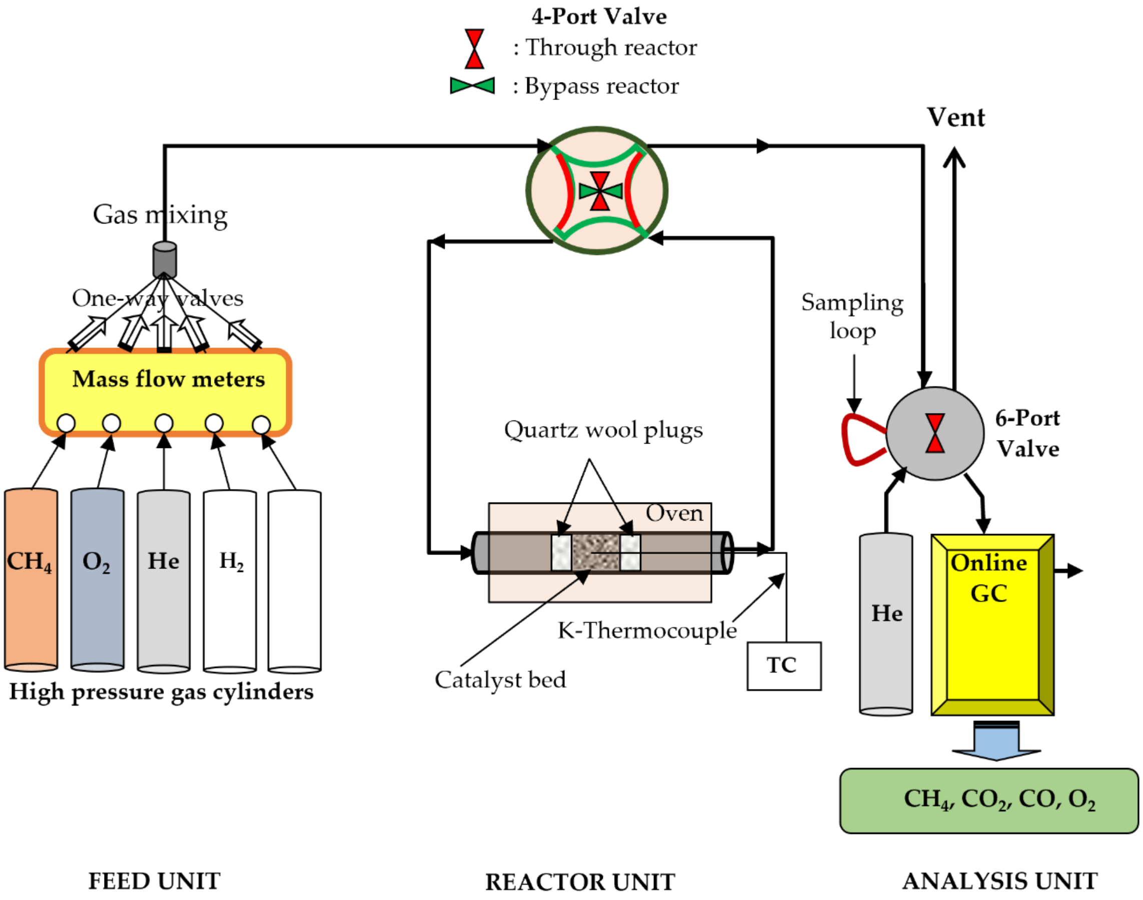

Figure S1. Schematic configuration of the continuous flow experimental apparatus equipped with on-line Gas Chromatography.

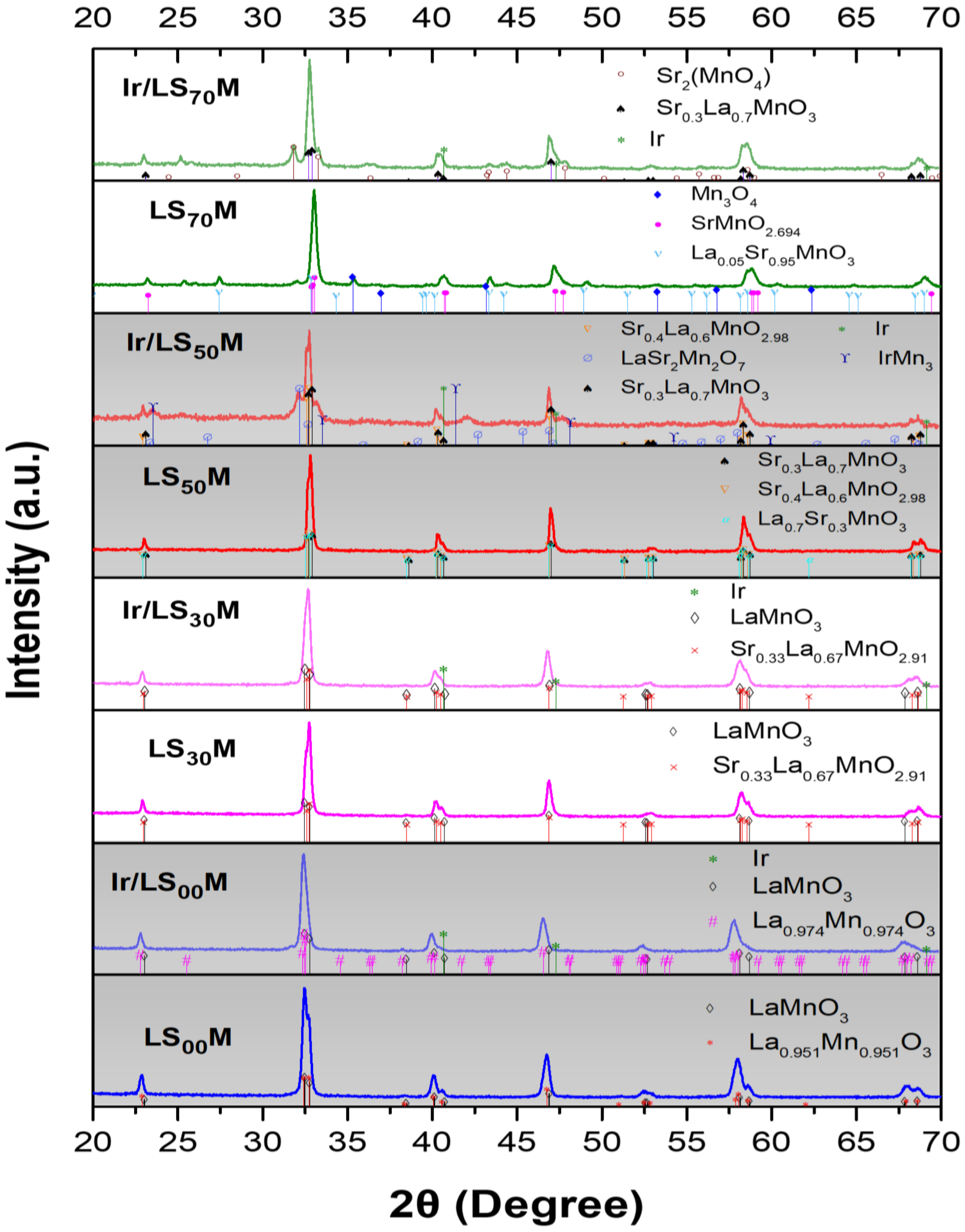

Figure S2. XRD patterns of LSxM perovskites ($La_{1-x}Sr_xMnO_3$; X = 0, 30, 50 and 70% substitution of La by Sr) and the corresponding 2 wt% Ir/LSxM catalysts.

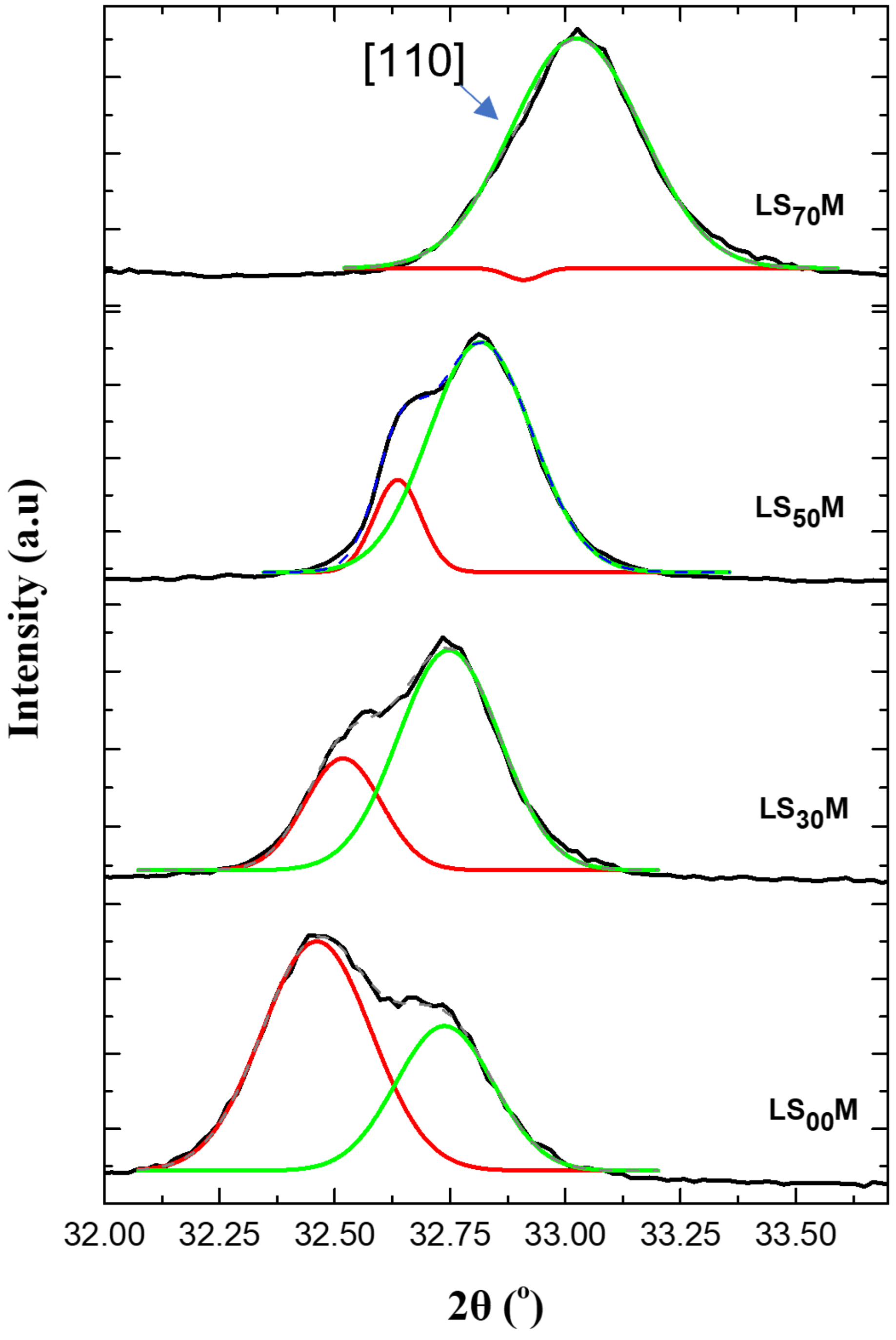

Figure S3. Magnification of the XRD patterns of LSxM perovskites at the region 2θ = 32 – 34°, where the main peak of the perovskite phase appears.

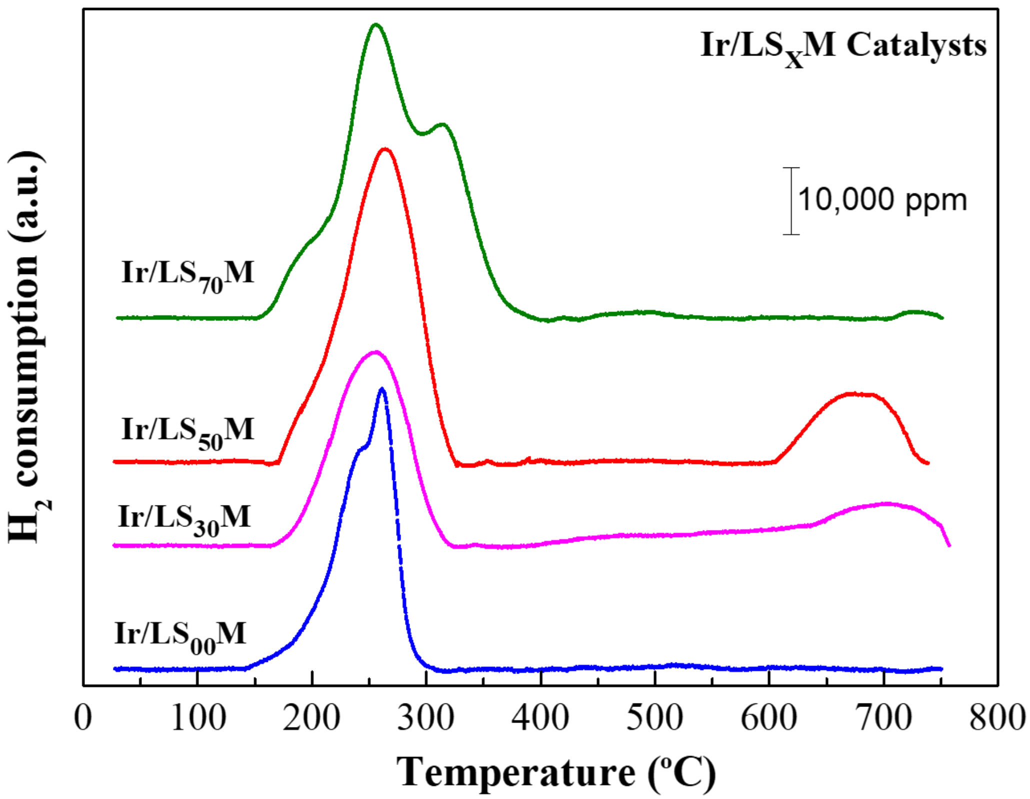

Figure S4. H_2 consumption versus temperature (H_2-TPR profiles) of Ir/LS$_X$M catalysts.

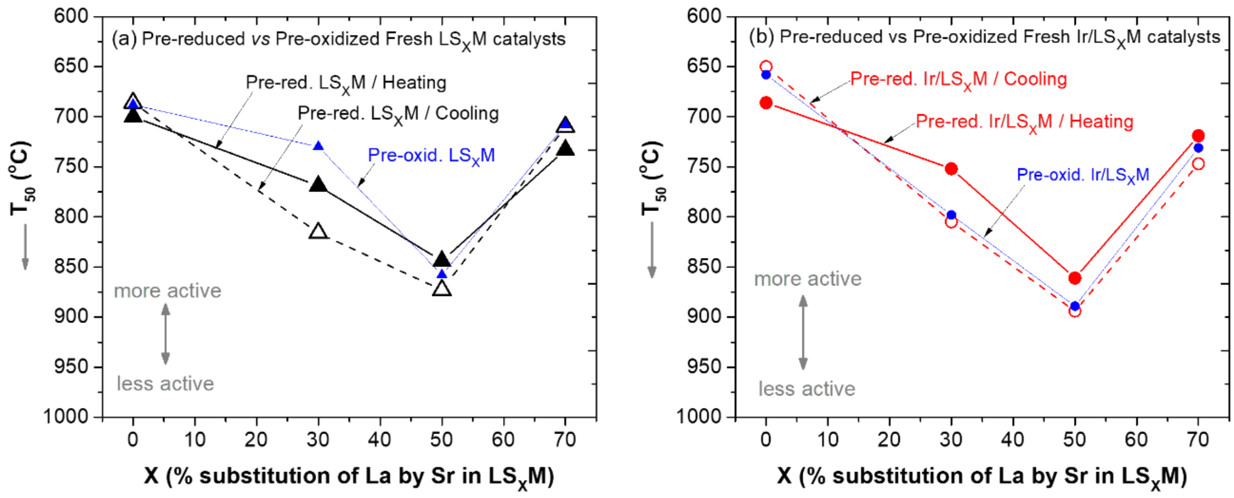

Figure S5. Dependence of T_{50} on X (% substitution of La by Sr in the perovskite composition) for pre-reduced and pre-oxidized LS$_X$M catalysts (a), and pre-reduced and pre-oxidized Ir/LS$_X$M catalysts (b). Feed conditions: 1.0% CH_4 + 5.0% O_2, He balance at 1atm, F_T=75 mL/min, m_{cat} = 50 mg, WGHSV=90,000 mL/g·h.

Supplementary Tables

Table S1. Temperature for 50% CH_4 conversion (T_{50}) of pre-oxidized and pre-reduced, *fresh* LS_XM and Ir/LS_XM catalysts.

Fresh Catalysts	T_{50} (°C) Pre-oxidized catalysts		T_{50} (°C) Pre-reduced catalysts	
	Light-off (heating)	***Light-out (cooling)***	***Light-off (heating)***	***Light-out (cooling)***
$LS_{00}M$	688	688	700	686
$LS_{30}M$	730	730	769	816
$LS_{50}M$	858	858	844	873
$LS_{70}M$	708	708	733	710
Ir/$LS_{00}M$	658	658	686	650
Ir/$LS_{30}M$	798	798	752	805
Ir/$LS_{50}M$	889	889	861	894
Ir/$LS_{70}M$	731	731	719	747

Table S2. Temperature for 50% CH_4 conversion (T_{50}) of pre-reduced and pre-oxidized LSxM and Ir/LSxM catalysts that were aged at 750 °C.

Aged@750 Catalysts	T_{50} (°C) Pre-oxidized catalysts		T_{50} (°C) Pre-reduced catalysts	
	Light-off (heating)	***Light-out (cooling)***	***Light-off (heating)***	***Light-out (cooling)***
$LS_{00}M$	681	681	699	687
$LS_{30}M$	838	838	813	840
$LS_{50}M$	897	897	877	898
$LS_{70}M$	717	717	737	727
Ir/$LS_{00}M$	657	657	687	653
Ir/$LS_{30}M$	842	842	769	830
Ir/$LS_{50}M$	912	912	895	913
Ir/$LS_{70}M$	752	752	715	751
